# Developmental warming induces severe deformities and mortality in a thermally tolerant fish species

**DOI:** 10.1038/s41598-026-39489-1

**Published:** 2026-03-26

**Authors:** Lucinda C. Aulsebrook, Taylor L. Hosler, Jennifer M. Donelson

**Affiliations:** College of Science and Engineering, James Cook University, Townsville, QLD 4814 Australia

**Keywords:** Climate change, Heat stress, Thermal tolerance, Skeletal development, Developmental plasticity, Gambusia holbrooki, Ecology, Ecology, Zoology

## Abstract

Climate change poses an unprecedented global threat to wildlife, with projected environmental warming likely to push many species beyond optimum temperature ranges. Understanding thermal limits is therefore critical for risk predictions and conservation management, especially in the context of sensitive life stages such as early development. We investigated the effects of elevated developmental temperatures on the eastern mosquitofish, *Gambusia holbrooki*. This species is a highly successful invader across temperate to tropical regions, making it an ideal model for understanding potential outcomes for more tolerant species. Wild mosquitofish were collected from tropical waterways and bred under their thermal optimum of 27 °C. Offspring were reared from birth across three temperature treatments: 27 °C – control, 30 °C – a higher temperature within their thermal range, and 33 °C, a temperature designed to test their thermal boundaries. Alarmingly, 84% of the fish exposed to 33 °C developed severe spinal deformities and there was 100% mortality by 135 days old. While spinal deformation was less common at 30 °C, there was significantly reduced survival by 220 days. Our study demonstrates the importance of studying critical life stages, including early development, to understand the thermal sensitivity of wildlife, as even species that are known to have highly robust adults may face detrimental outcomes in climate change scenarios.

## Introduction

Climate change is a global threat to ecosystems, with a 1.4–2 °C increase in average temperature predicted by 2050^1^, as well as more frequent heat events exceeding current temperatures by 5 °C or more^2^. Such increases in temperature are expected to put many species beyond their historical thermal range and performance limits^3,4^, which could lead to dramatic population declines or extinctions. Ectotherms, such as fish, reptiles and amphibians, are particularly vulnerable to temperature changes, as they do not have internal temperature regulation, and accordingly cellular processes are controlled by environmental temperature^5,6^. Consequently, temperature plays a critical role in many vital functions, including physiological processes, morphology and behaviour^7^. Indeed, elevated temperature has been shown to shift many key traits across ectotherm species, such as increasing metabolism^6,8–11^, reducing body size^12,13^, disrupting reproduction^6^ and altering behaviours such as shoaling and predator evasion^14^. Such disruptions can reduce survival and fitness at elevated temperatures, contributing to the global decline of ectotherms^15,16^.

Thermal conditions during early development can be particularly influential on adult phenotypes, since early life stages are highly sensitive to environmental conditions^17,18^. Sensitivity during early life may be especially influential because epigenetic changes during early development can impact a higher proportion of cells^19^. Deviation from optimal temperatures in these early stages can disrupt the development of traits, sometimes with maladaptive and permanent consequences^20–22^. For example, an increase of 3–4 °C above optimal temperatures during incubation or early development can negatively affect skeleton formation and swimming performance in gilthead seabream^23^, the ability for turtles to self-right, crawl and swim^24^, and locomotor performance and sperm development in delicate skinks^25^. On the other hand, early exposure to altered conditions can potentially benefit individuals through inducing adaptive or beneficial phenotypic changes in response to environmental conditions^17,26^. For example, zebrafish exposed to 5 °C below or above their optimum during early development, but not throughout life, have been found to have improved swimming performance at those same temperatures in later adult life stages^27^. Environmental warming during early development thus has the potential to strongly influence adult phenotypes and performance, both negatively and positively.

Assessing the responses of ectotherms to elevated temperatures, particularly throughout critical developmental stages, is essential for understanding the future consequences of climate change on ecosystems. For most species, we have a limited understanding of how future projected warming and extreme events relate to species’ current thermal optimum and thresholds for performance. Yet, these attributes, along with plasticity, will determine the severity of the impact of climate change on populations and species^28,29^. To better predict and understand the response of ectotherms to climate change, there is a strong need for experimental studies to determine critical thermal thresholds, and verify clear causal links between elevated temperatures and individual level physiological impacts, thus increasing the accuracy of predictions at a level suitable for wildlife management^30^.

Here, we experimentally investigated the effect of increased developmental temperature on eastern mosquitofish (*Gambusia holbrooki*), a robust live-bearing model species known to inhabit a wide thermal range of 8–38 °C^31,32^. Previous research on early developmental temperature exposure is limited in this species, but has found that higher temperatures tend to result in faster maturity and smaller body size^32–34^. These studies, however, are limited to a maximum of 32 °C and 10 weeks of exposure, and do not examine effects on survival^33^. In this study, we exposed mosquitofish from birth to three rearing temperature treatments: 27 °C – their thermal optimum, 30 °C – a higher temperature within their thermal range, and 33 °C, a temperature expected to be moderately stressful. The development and survival of the offspring were then monitored for the duration of 220 days, and any deceased individuals were preserved for observation of developmental abnormalities. Surprisingly, even in this resilient species, we found dramatic malformation and mortality at elevated temperatures, raising alarming insights into the future of less tolerant species.

## Methods

All experimental procedures were approved by James Cook University Animal Ethics Committee (JCU A2930) and were performed in accordance with the Australian Code for the Care and Use of Animals for Scientific Purposes. Fish collection and use were authorised under biosecurity permit PRID001014 from the Queensland Government Department of Agriculture and Fisheries, and permit 270220 from Fisheries Queensland. The study is reported in accordance with ARRIVE guidelines (https://arriveguidelines.org/).

### Study species

The eastern mosquitofish (*Gambusia holbrooki*) is a tropical freshwater live-bearing fish native to North America^35^. It was introduced to Australia in 1925 to control mosquitoes but is now highly invasive^36^ due to its hardiness^37^ and tolerance to a broad spectrum of environments. Throughout the native and invasive range globally, populations inhabit water temperatures from a minimum of 8 °C^32^, to a maximum of 38 °C^31,35^. Eastern mosquitofish breed throughout the year provided that the water temperature is above 15 °C^38^, although studies have indicated that temperatures above 32 °C can impair growth, condition and swimming performance^33,34^. Research has yet to establish an upper thermal limit for reproduction, but males have been observed to engage in copulatory behaviour at temperatures as high as 38 °C^31^. The thermal optimal of eastern mosquitofish in Queensland is considered to be around 27 °C, based on their population distribution, and peak adult reproductive and swimming performance^31^.

### Fish collection and housing

Wild mosquitofish were collected in May to June 2024 from four waterways across Townsville: Lake Idalia (-19.308, 146.816), Mundy Creek (-19.257, 146.785), Paradise Lake (-19.272, 146.789) and Bushland Beach (-19.203, 146.674). Within 3 h of collection, fish were transported to our aquarium facilities where each fish was housed individually in a 10 L tank (dimensions 23 × 23 × 31 cm) with a flow through system that utilised municipal water, dechlorinated via activated carbon. Water quality tests were conducted to ensure suitability of water parameters, including pH, general hardness, alkalinity and ammonia, nitrite and nitrate levels. Each tank contained constant gentle aeration, and fish were provided with a PVC tube (3.5–5 cm diameter) as a refuge and flyscreen attached to simulate plant structure. Tanks were kept in a 12:12 h light-dark cycle and in tank temperatures were maintained at 27 °C (± 0.4 °C) through a heat pump (Toyesi TAC 600 SSD) in the main system sump. All fish were fed daily ad libitum with INVE Aquaculture NRD 5–8 mm dry pellets (54% crude protein and 13% crude fat).

### Breeding and offspring treatments

Wild-caught females were randomly paired with a male collected from the same site for breeding. The paired male was added to the female tank for two weeks before being returned to his individual tank. Females were then checked daily for offspring, and offspring from each clutch were collected and evenly split across the three temperature treatments: 27 °C, 30 °C and 33 °C, with random assignment of any remainder offspring. This split-clutch design controlled for genetic background, transgenerational effects, embryonic conditions and variation in birth timing across temperature treatments. Offspring were placed into 2 L containers filled with natal tank water and acclimated through gradually adding water of their assigned temperature treatment, before being moved to a 10 L tank in their treatment. Temperature treatments were maintained by thermostat heaters in each system’s sump, with random tank thermometer testing showing treatment averages and standard deviations of 26.9 ± 0.2 °C, 30.0 ± 0.2 °C and 32.7 ± 0.5 °C respectively.

Overall, 19 clutches of 6–19 offspring were produced, resulting in approximately 50 offspring in each temperature. Final replicates included 29 individuals in the 27 °C treatment, 36 in 30 °C and 35 in 33 °C, where differences were due to early mortality (not identifiable due to bodies degrading too quickly), as well as a few fish escaping their tanks into the water reservoir. Sixty-four individuals were produced from Mundy Creek pairs, 18 from Bushland Beach, 12 from Lake Idalia and 6 from Paradise Lake. Siblings of the same clutch, age and temperature treatment were housed together in 10 L tanks until maturity, whereupon males and females were separated. All individuals were fed ad libitum daily, and any mortality recorded.

On 13th September 2024, when juveniles were 6–10 weeks old, a number of deformities were noted in individuals in the 33 °C treatment. From this point, any individuals found deceased were removed and preserved for clearing and staining (details below) to examine skeletal deformities. Additionally, a selection of individuals were euthanised and sampled on the same day (16/09/2024) to allow comparisons of developmental morphology across all source populations and temperature treatments at the same life stage. Specifically, at least one deformed individual from each source population was selected to be euthanised and preserved for clearing and staining. For each of these individuals, a sibling from 27 °C to 30 °C was also euthanised for staining and clearing on the same day. This resulted in a total of 5 individuals from each temperature treatment being euthanised. Following this initial identification of skeletal issues, fish were checked daily and any individuals that had issues swimming or maintaining buoyancy were euthanised by an overdose of clove oil and preserved for clearing and staining.

### Staining and angle measurement

**Fig. 1:**
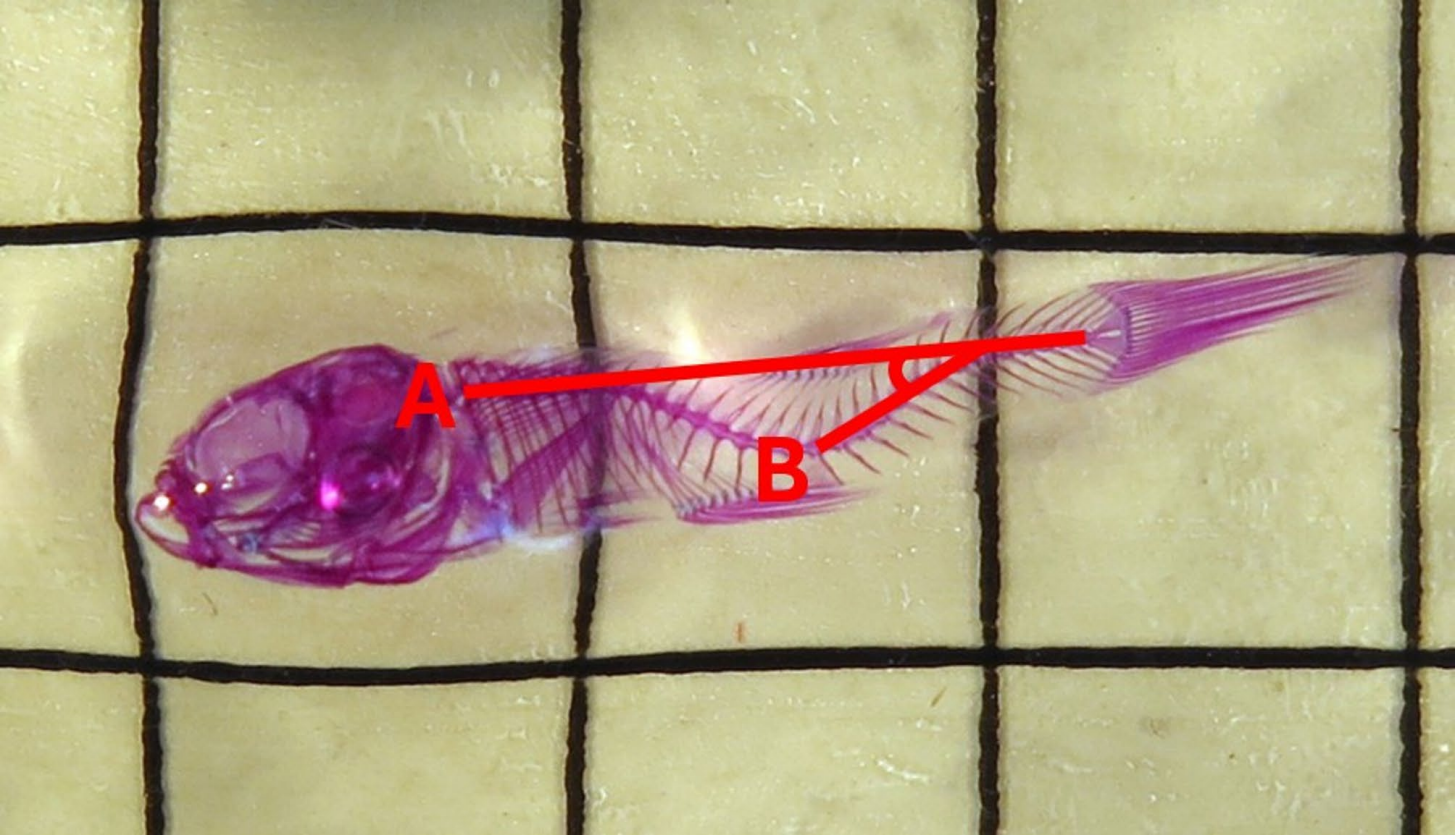
An example measurement of the spinal curvature on a cleared and stained *Gambusia holbrooki* specimen. The angle was measured between (A) a line connecting the first and last segment of the spine and (B) a line along the section of the spine that deviated furthest from line A. Individuals were categorised as deformed if the measured angle was greater than 15°. The depicted specimen has a spinal curvature angle of 31.6°.

Specimens were fixed in 10% neutral buffered formalin for 24 h and then transferred to 70% ethanol for preservation. Clearing and staining was conducted following the procedure of Taylor and Van Dyke^39^, using alcian blue and alizarin red for staining cartilage and bone, and trypsin for clearing tissue. Following staining and clearing, all specimens were photographed, and spine angles were measured on ImageJ (version 1.54). Spine angle was determined using same method as Wang and Tsai^40^, where the angle measured was between a line connecting the first and last segment of the spine, and a line along the section of the spine that deviated furthest from the first line (Fig. 1). Similarly to their study, individuals were classified as deformed if the curvature angle was greater than 15°.

### Measurement of environmental temperatures

To measure the upper thermal range of the sampled mosquitofish populations, temperature data was obtained from the collection sites from January 9th to 31st 2025. Two HOBO UA-002-08 temperature data loggers were placed at each site and the water temperature was logged every 10 min. Loggers were placed 1–2 m from the water’s edge at depths within 2 m, since mosquitofish have a preference for shallow waters^37^. Data collected from the same site was combined when calculating mean and maximum temperatures, as well as proportion of time above 30 °C and 33 °C. All four waterways that mosquitofish were sourced from were found to have average temperatures from 30 to 33 °C across January 2025 (Table 1). In the lake environments (Lake Idalia and Paradise Lake), temperatures regularly exceeded 33 °C (50–60% of the total time measured), reaching levels as high as 40 °C. In the creek environments (Bushland Beach and Mundy Creek), temperatures only exceeded 33 °C at the hottest times of the day (4–6% of the total time measured), but still had an overall average of 30 °C.

**Table 1:** Average and maximum summer temperatures of waterways that mosquitofish were sourced from, as well as percentage of time above 30 °C and 33 °C. Temperature data was collected from January 1st to 31st 2025 through two HOBO UA-002-08 temperature data loggers placed in the water of each site.

Waterway	Average temperature	Maximum temperature	Time above 30 °C	Time above 33 °C
Bushland Beach	29.9 °C	35.1 °C	47.9%	6.0%
Lake Idalia	33.1 °C	37.9 °C	88.5%	60.5%
Mundy Creek	30.3 °C	33.7 °C	66.1%	4.5%
Paradise Lake	32.3 °C	40.5 °C	78.4%	50.0%

### Statistical analysis

Statistical analyses and figure generation were conducted using R: A Language and Environment for Statistical Computing (version 4.4.0, https://www.R-project.org/). To determine the effects of temperature on spine angle, we used a linear mixed effect model from the lme4 package^41^, using temperature as a fixed effect, and sibling group as a random effect. Spine angle data was log-transformed (natural log) prior to analysis to adhere to assumptions of normality and homogeneity of variance. Differences between temperature treatments were determined using a Type III Wald chi-square test applied to the model. Where significant differences were found, post-hoc pairwise comparisons were conducted using the emmeans package^42^ with Tukey’s adjustment for multiple comparisons. Complete separation prevented fitting logistic mixed effect models for deformity incidence per temperature treatment; therefore differences in the proportion of deformed individuals were analysed using a chi-squared test, and a pairwise fisher’s test^43^ was used for post-hoc analysis. Survival analysis was conducted using a mixed effect cox regression^44^, using temperature as a fixed effect and sibling group and source population as random effects. Euthanised individuals were omitted from survival analysis, resulting in sample sizes 24 individuals at 27 °C, 32 at 30 °C and 30 at 33 °C. Tukey’s post-hoc analysis was conducted with the emmeans package. Figures were generated using ggplot^45^ and survminer^46^.

## Results

### Elevated temperature increased the frequency and severity of spinal deformities

**Fig. 2:**
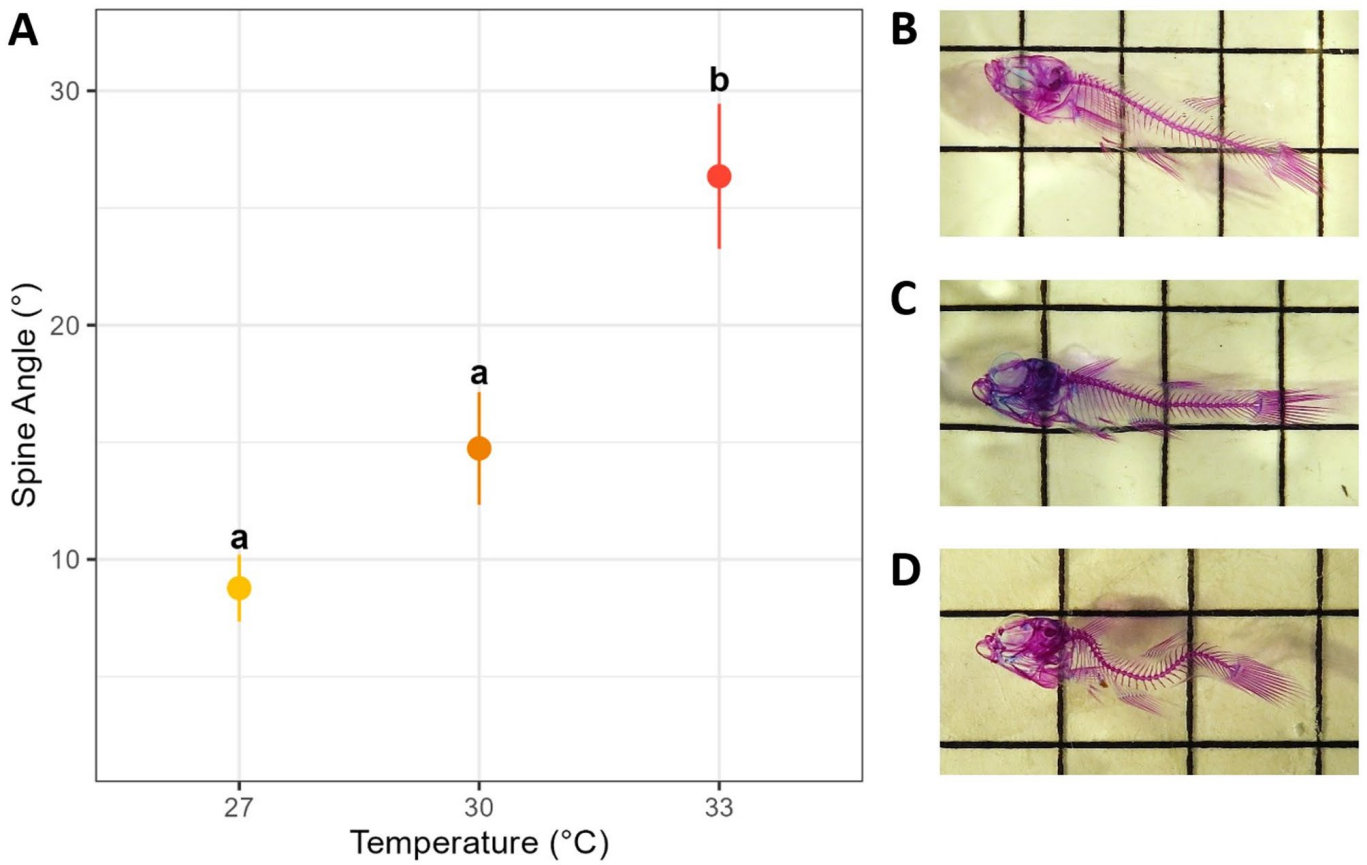
The effect of developmental temperature on spinal curvature. (**A**) Points represent raw (observed) mean angle of spinal for each temperature group (± SE) and lowercase letters indicate significant groupings from post-hoc analysis. (**B**) An example specimen from the 27 °C treatment after clearing and staining, where alizarin red was used to stain the bones, compared to (**C**) a cleared and stained specimen from 30 °C treatment and (**D**) a similar specimen from the 33 °C. All three depicted specimens were born in the same clutch and euthanised on the same day.

We found that post-natal developmental temperature had a significant effect on the incidence of deformity (χ^2^ = 47.331, df = 2, *p* < 0.001) and the severity of deformity (angle: χ^2^ = 156.280, df = 1, *p* < 0.001) in eastern mosquitofish. The 33 °C treatment resulted in 86% of fish developing spinal malformations, whereas no incidents of deformity were observed in the 27 °C treatment (*p* < 0.001, Table 2). The severity of spinal curvature was also most extreme at 33 °C with an average angle of 26.3° (Fig. 2). Although 18% of fish in the 30 °C treatment developed abnormally, this level of incidence was not significantly different from 27 °C (*p* = 0.07), and their average spinal angle of 14.7° was not significantly different from the 27 °C average of 8.8° (*p* = 0.432, Fig. 2).

**Table 2:** Occurrence of spinal deformities in each temperature treatment. Fish were defined as being deformed if the angle of their spinal curvature was greater than 15°. Fish that died before 13th September 2024 were not preserved for analysis and were omitted from the total. Of the fish with spinal deformities, 5 from 33 °C were euthanised and all others died naturally

Temperature treatment	Total number of fish	Number of fish with spinal deformities
27 °C	29	0
30 °C	28	5
33 °C	22	19

### Survival probability decreased with increasing temperatures

**Fig. 3:**
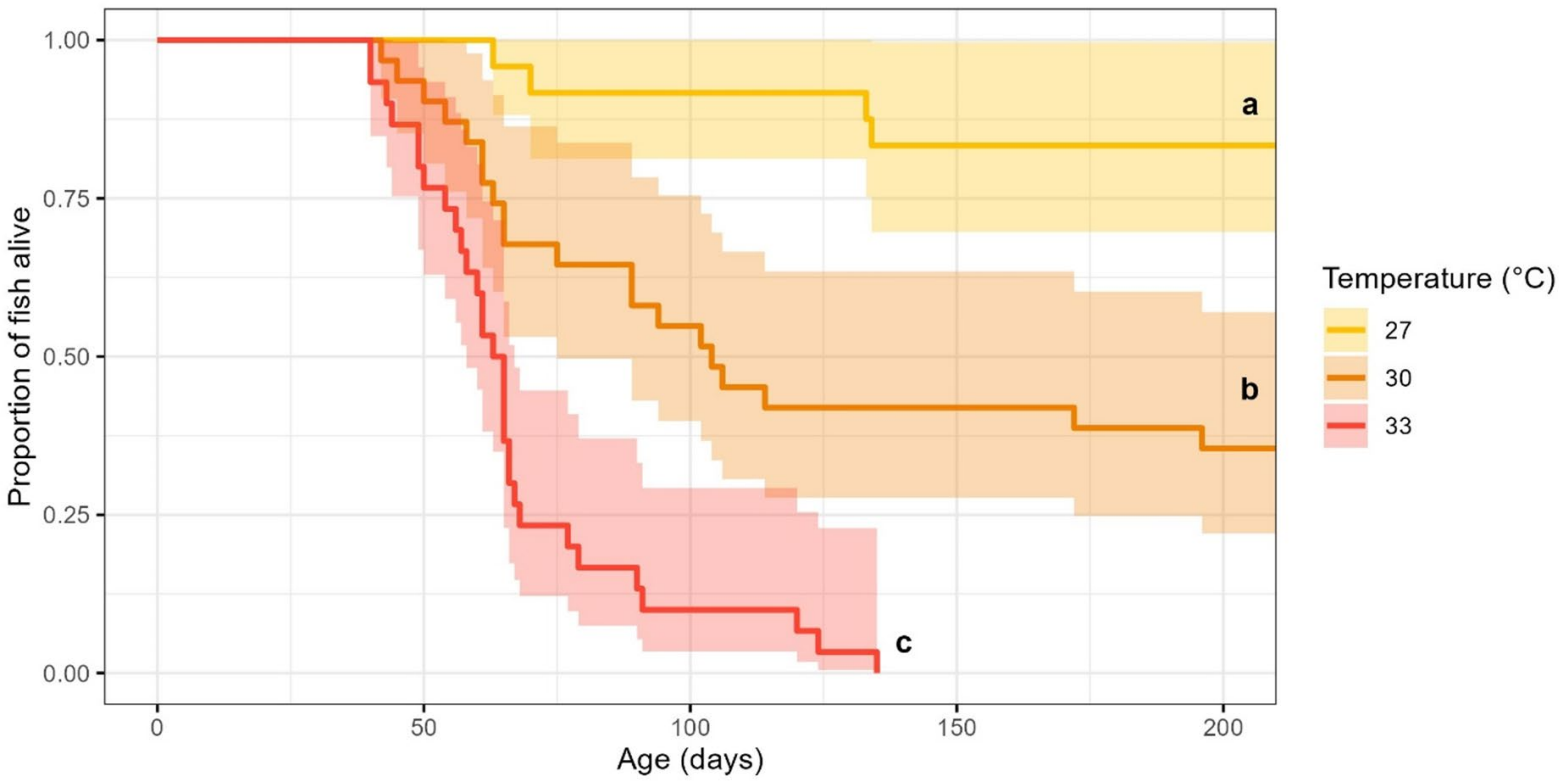
Proportion of fish alive within each temperature treatment over the course of 220 days. Shading represents 95% confidence intervals and lowercase letters indicate significant groupings from post-hoc analysis.

Some mortality was seen in all temperature treatments from 40 days post-birth, but survival rate was significantly influenced by developmental temperature (χ^2^ = 30.823, df = 2, *p* < 0.001). Specifically, fish exposed to 33 °C had substantially lower survival than those in cooler temperature treatments (both *p* < 0.001), with less than 25% remaining at 68 days post-birth and none remaining by day 135. Fish that developed in 30 °C initially showed similar mortality to 33 °C from 40 to 60 days, resulting in lower survival than 27 °C (*p* = 0.030), but at 220 days 38% remained alive (Fig. 3).

## Discussion

Knowledge of where species are currently living in relation to their thermal limits is critical for predicting future ramifications of climate change. We found that exposure from birth to 33 °C impacted spinal development of eastern mosquitofish, and developmental temperatures of 30–33 °C reduced survival rate. In contrast, previous studies that held adult mosquitofish at 30–32 °C for six weeks^47,48^ or acutely exposed adults to 32–38 °C^31,49^ have found minimal negative effects, and healthy wild populations have been observed in habitats that range between 30 and 35 °C^35,50^. Indeed, temperature loggers in the waterways from which we sourced our parental fish revealed an average temperature of 30–32 °C through summer, with temperatures above 33 °C for 50–60% of the day in two of the habitats measured. Our findings therefore indicate that while temperatures above 30 °C are tolerable for adult mosquitofish, early development stages are far more sensitive to elevated temperature, which may be unsurprising considering its rapid development, reaching maturity as early as 40 days^35^.

Our study revealed that elevated developmental temperature severely affects spinal formation of eastern mosquitofish, with 86% of fish exposed to 33 °C displaying some level of spinal deformity, in contrast to zero incidents at 27 °C. Very few studies have examined skeletal development in mosquitofish, although Hubbs^51^ noted that western mosquitofish specimens collected from warmer springs (around 35 °C) had a higher incidence of vertebral deformities, and Sassi et al.^52^ found that exposure to cadmium and 32 °C resulted in a higher incidence of skeletal deformities compared to 24 °C. To our knowledge, this study is the first to show an association between temperature and spinal development in the eastern mosquitofish. Nonetheless, spinal deformities have been observed in a number of other fish species when larvae are reared 3–5 °C above optimal temperatures, such as golden pompano^53,54^, clownfish^55^, and European sea bass^56^. These studies are generally targeted towards informing ideal temperatures for fish cultivation and farming, rather than in the context of climate change. Notably, in studies where three or more temperatures were compared, the incidence of spinal deformities appeared to dramatically increase once a certain temperature threshold was reached (33 °C for golden pompano and 34 °C for clownfish), similar to the increase seen between 30 °C and 33 °C in our study.

While the exact mechanism behind the association between elevated developmental temperature and spinal malformation is yet to be determined, a potential explanation is that the rapid development and growth rate associated with increased temperatures^9^ can lead to growing muscles exerting high pressure on developing bones if the temperature is above a certain threshold^54–57^. Indeed, elevated temperature during development has been demonstrated to result in changes in muscular growth, such as the formation of larger muscle fibres, in a variety of fish species^58–60^. Additionally, many transcription factors and signalling molecules are regulated by temperature, which could also contribute to developmental abnormalities^54^. Given that abnormal skeletal development in response to increased developmental temperature has been observed in a diverse range of fish families, such an association is likely to occur in many other fish species and should be considered when predicting the consequences of climate change on fishes.

In addition to increasing the occurrence of spinal deformity, higher temperatures also reduced the survival of mosquitofish. This could be related to the spinal deformities observed, as severe malformation can negatively affect the swimming and foraging ability of fish^61,62^. Indeed, many studies have confirmed reduced survival in individuals with spinal deformities^54,62,63^, although in some cases spinal deformities do not appear to prevent fish from maturing and reproducing^51^. However, increased mortality (by 41%) was also observed in fish that developed at 30 °C, which did not have a significant increase in deformity. Further, of the fish that died in 30 °C, only 25% were found to be deformed. Increased temperature may therefore reduce survival regardless of skeletal malformations, perhaps due to the increased metabolic costs, reduced appetite or overall stress associated with higher temperatures^64^. It is likely that any impacts to performance from warming in a natural setting would have greater ramifications than observed in our experiment, since our estimates are in the absence of natural processes such as competition and predation. Additionally, our study did not include thermal exposure during the gestational stage, which could further exacerbate the impacts we observed due to the sensitivity of this early developmental period, although it is also possible that embryonic exposure could improve thermal tolerance through beneficial transgenerational or developmental plasticity, as demonstrated in a variety of fish species (e.g^65–68^.

Given the wide thermal tolerance and hardiness of eastern mosquitofish, the extreme responses to elevated temperatures our study found were unexpected, but likely give a reliable indication of critical thermal limits. In the wild, it is possible that juveniles have hindered development or are unable to survive in the hotter months of the year, but populations are unlikely to be impeded due to mosquitofish breeding throughout the year^35^. With projected global temperature increases, however, the colder parts of the year may more regularly exceed 33 °C, which could result in population declines. This sets a worrying precedent for the survival of less tolerant, native species in the context of climate change, especially given that associations between elevated temperatures and skeletal deformities have been observed in a diverse range of species. If a general trend, the projected temperature increases may result in significantly higher rates of skeletal abnormality among many fish species, impeding their survival. In addition to ecological ramifications, this has critical implications for food security with fish a critical protein source for many human populations^69,70^.Our study therefore emphasises the strong need to understand the impacts of environmental change with chronic exposure and across life stages, and evaluate which species are currently at their thermal limit, in order to inform mitigation strategies and targeted conservation efforts.

## Data Availability

The data that support the findings of this study are openly available in figshare at 10.6084/m9.figshare.31238707.
